# A Quantitative Framework to Identify and Prioritize Opportunities in Biomedical Product Innovation: A Proof-of-Concept Study

**DOI:** 10.1001/jamahealthforum.2023.0894

**Published:** 2023-05-05

**Authors:** Laura Elisabeth Gressler, Kenyon Crowley, Elise Berliner, Hartley Leroy, Esther Krofah, Benjamin Eloff, Danica Marinac-Dabic, Meena Vythilingam

**Affiliations:** 1 Center for Devices and Radiological Health, US Food and Drug Administration, Silver Spring, Maryland; 2 University of Arkansas for Medical Sciences, Little Rock; 3 Robert H. Smith School of Business, University of Maryland, College Park; 4 Accenture Federal Services, Arlington, Virginia; 5 Cerner Enviza, Kansas City, Missouri; 6 Faster Cures and Center for Public Health, Milken Institute, Washington, DC; 7 Office of the Assistant Secretary for Health, US Department of Health and Human Services, Washington, DC

## Abstract

**Importance:**

Prioritization and funding for health initiatives, including biomedical innovation, may not consistently target unmet public health needs.

**Objective:**

To (1) develop a quantitative, databased framework to identify and prioritize opportunities for biomedical product innovation investments based on a multicriteria decision-making model (MCDM) that includes comprehensive measures of public health burden and health care costs, and (2) pilot test the model.

**Design, Setting, and Participants:**

The Department of Health and Human Services (HHS) convened public and private experts to develop a model, select measures, and complete a longitudinal pilot study to identify and prioritize opportunities for investment in biomedical product innovations that have the greatest public health benefit. Cross-sectional and longitudinal data (2012-2019) for 13 pilot medical disorders were obtained from the Institute for Health Metrics Global Burden of Disease database (IHME GBD) and the National Center for Health Statistics (NCHS).

**Main Outcome Measures:**

The main outcome measure was an overall gap score reflecting high public health burden (composite measure of mortality, prevalence, years lived with disability, and health disparities), or high health care costs (composite measure of total, public, and out-of-pocket health spending) relative to low biomedical innovation. Sixteen innovation metrics were selected to reflect the pipeline of biomedical products from research and development to market approval. A higher score indicates a greater gap. Normalized composite scores were calculated for public health burden, cost, and innovation investment using the MCDM Technique for Order of Preference by Similarity to Ideal Solution method.

**Results:**

Among the 13 conditions tested in the pilot study, diabetes (0.61), osteoarthritis (0.46), and drug-use disorders (0.39) had the highest overall gap score reflecting high public health burden, or high health care costs relative to low biomedical innovation in these medical disorders. Chronic kidney disease (0.05), chronic obstructive pulmonary disease (0.09), and cirrhosis and other liver diseases (0.10) had the least amount of biomedical product innovation despite similar public health burden and health care cost scores.

**Conclusions:**

In this cross-sectional pilot study, we developed and implemented a data-driven, proof-of-concept model that can help identify, quantify, and prioritize opportunities for biomedical product innovation. Quantifying the relative alignment between biomedical product innovation, public health burden, and health care cost may help identify and prioritize investments that can have the greatest public health benefit.

## Key Points

**Question:**

Can a data-driven, quantitative approach help identify and prioritize opportunities in biomedical product innovation for medical disorders with high public health burden and high health care costs?

**Findings:**

In this cross-sectional pilot study of 13 conditions, a quantitative multicriteria decision-making model that evaluated the extent of biomedical product innovation relative to comprehensive measures of public health burden and health care costs identified diabetes, osteoarthritis, and drug-use disorders as having the highest overall gap score reflecting high public health burden, or high health care costs relative to low biomedical innovation in these medical disorders. Chronic kidney disease, chronic obstructive pulmonary disease, and cirrhosis and other liver diseases had the least amount of biomedical product innovation despite similar public health burden and health care cost scores.

**Meaning:**

By analyzing the relative alignment between quantitative measures of biomedical product innovation, public health burden, and health care costs, this data-driven, quantitative model can help identify and prioritize investments that can have the greatest public health benefit.

## Teaser

This proof-of-concept study examines whether a data-driven, quantitative approach can work to help identify and prioritize opportunities in biomedical product innovation for medical disorders with high public health burden and high health care costs.

## Introduction

Prioritization and funding for health initiatives, including biomedical innovation, may not consistently target unmet public health needs. Public health leaders and industry funders who rely on select metrics like mortality to inform strategic priorities and funding decisions can potentially overlook important considerations, including the effects of disability, cost of treatment, and health disparities. Furthermore, medical research priorities are frequently determined by expert consensus or stakeholder ranking^1,2^ and can be influenced by advocacy for or bias against select medical disorders.^3^ A quantitative framework with comprehensive metrics that leverages latest or currently available data across the health ecosystem can help identify, track, prioritize, and align opportunities in biomedical product innovation to address unmet health needs in communities.^4,5^

Few studies have analyzed comprehensive health data from diverse domains to identify gaps in focused areas such as research and innovation.^6,7,8,9^ Rees et al^6^ evaluated mortality, disability-adjusted life-years (DALY), and health care use for pediatric medical conditions and reported a positive correlation between National Institutes of Health (NIH) funding for pediatric research and only some, but not all, metrics of pediatric disease burden. Research evaluating funding allocation by cancer type found that funding was moderately proportional to the incidence of the cancer type and was poorly correlated with deaths and potential years of life lost (PYLL).^10^ A Healthcare, Technology, and Innovation for Economic Success (HealthTIES) consortium in Europe developed indicators and indexes to measure and benchmark the extent of innovation in medical and life sciences across academia, industry, and government.^7^ Additional models such as the Navigating the Ecosystem of Translation Sciences (NETS)^11^ and the Drug Discovery, Development, and Deployment Map (4DM)^12^ accounted for the complex interactions in the innovation ecosystem and included metrics that measured the pipeline of biomedical products from research and development to market approval.^13^ To date, studies have not successfully integrated and simultaneously analyzed metrics from complementary domains, including public health burden, cost, and biomedical product innovation, to identify and prioritize opportunities for investment in biomedical product innovation.

In the backdrop of a resource-intensive COVID-19 pandemic, the Office of the Assistant Secretary for Health, Department of Health and Human Services (HHS) convened experts from the federal government, academia, industry, and the nonprofit sector to (1) develop a quantitative, data-driven framework to identify, track, and prioritize opportunities for biomedical product innovation for medical disorders with the highest public health burden and health care cost and (2) pilot test the model in select medical disorders.

## Methods

For this longitudinal study, a multidisciplinary team of epidemiologists, physicians, economists, engineers, businesspersons, and public health professionals developed a framework to define biomedical product innovation opportunities, select measures, identify data sources, and design an analytic strategy for the pilot study. Biomedical product innovation opportunities were defined as medical conditions in which the extent of innovation activity was relatively low compared with the high public health burden and higher health care costs. Three domain-specific working groups were tasked to develop definitions and identify US-specific metrics and data sources to assess and quantify public health burden, health care costs, and the extent of biomedical product innovation, respectively. All 3 working groups met periodically as a larger work group to collaborate, receive feedback, and integrate data from all 3 domains to inform the final framework and pilot study. This study was deemed exempt from written informed consent because all data used were publicly available.

### Public Health Burden Metrics

Public health burden was assessed by analyzing 4 related measures including mortality and years lived with disability (YLD) sourced from the Institute for Health Metrics Global Burden of Disease database (IHME GBD),^14^ prevalence data sourced from the National Center for Health Statistics (NCHS),^15^ and measures of health disparity calculated by using the Healthy People 2020 disparity summary rate ratio with sex, age, and racial and ethnic distribution for the mortality data provided by NCHS.^16^ Health disparities are defined as preventable differences in the burden of disease or opportunities to achieve optimal health experienced by socially disadvantaged populations. Health disparities were calculated by taking the maximum ratio of the most favorable or least adverse group rate and the average rate of all other groups.^16,17^ In addition to evaluating cross-sectional mortality, prevalence, and YLD in 2019 for all age groups, longitudinal trends in public health data between 2015 to 2019 were included in the model.

### Health Care Cost Metrics

Health care cost measures selected were total public spending, out-of-pocket (OOP) health expenses, and total spending and were extracted from IHME. It identifies government domestic, corporation, and nonprofit institution resources allocated for health and social insurance contributions using the World Health Organization’s (WHO) Global Health Expenditure Database (GHED).^18^ The IHME’s methods for calculating health care cost measures are described in further detail elsewhere.^19^ Total public spending data reflected the sum of Medicare and Medicaid costs as well as spending on public health system infrastructure. Out-of-pocket health spending encompassed payments made by individuals at or after the time of health care delivery, including copayments or deductibles. Health insurance premiums were not included in the OOP spending metric. Total spending was the sum of public spending, private health spending, and OOP health spending and did not include indirect health spending, such as lost wages due to illness or transportation costs. All health care cost measures were sourced from IHME. In addition to the cross-sectional health care cost metrics for 2016, changes in costs for total spending, total public spending, and OOP spending were calculated from 2012 to 2016. This allowed for an examination of the longitudinal changes in health care costs over time for the medical disorders evaluated in the pilot study.

### Biomedical Product Innovation Metric

For the purposes of this model, the extent of biomedical product innovation activity was defined as the volume and rate of advancement, development, and deployment of novel technologies, devices, diagnostics, and pharmacotherapies that have the potential to influence critical health-related and economic outcomes including disease prevalence, severity, quality of life, disparities in access, and the economic burden of disease. Through a review of the literature and expert input, the group generated more than 2 dozen measures to quantify the extent of innovation for a medical condition. These innovation measures were mapped to accessible data sources, and measures that represent parsimony among a singular concept (eg, clinical trial activity) were identified. The data were assessed qualitatively based on each possible measure’s accessibility, face validity, usability, and usefulness. Overall, 6 innovation measure categories (with data sources) comprising 16 metrics were selected: patent applications and patents granted (Google Patents with USPTO)^20^; federal investment dollars and projects (NIH RePORTER)^21^; private investment dollars and deals by early or late stage (Pitchbook)^22^; clinical trials started, ended, and those with results posted (ClinicalTrials.gov)^23^; and US Food and Drug Administration (FDA) number of approvals for drugs, devices, and expedited pathways; private investment9; and venture capital funding in dollars and deals by early or late stage (Pitchbook). Each of these 16 metrics was quantified annually from 2015 to 2019 to analyze cross-sectional and longitudinal changes in innovation activity.

### Overall Gap Calculation

**Figure 1.**
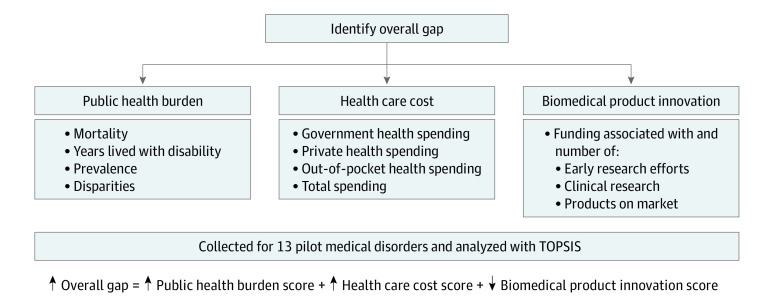
Model and Methodology to Identify Overall Gaps An overall gap score for each medical condition was calculated and reflects the relative ranking compared with 1 extreme alternative (lowest possible score: a theoretical condition with the lowest public health burden, lowest cost, and highest innovation level) and the other extreme possible alternative (highest possible score: a theoretical condition with the highest public health burden, highest cost, lowest innovation level). Low scores indicate an imbalance with relatively high investment in innovation relative to public health burden and cost, whereas high scores indicate an imbalance with relatively low investment in innovation relative to public health burden and cost. TOPSIS indicates Technique for Order of Preference by Similarity to Ideal Solution.

We piloted the method on a small set of medical conditions that were likely to have high public health burden. We first compiled 3 lists of the top 10 medical conditions with the highest mortality, prevalence, and YLD metrics, respectively, based on 2019 IHME and NCHS data. Out of the 30 medical conditions, the top 13 were selected after removing duplicates that appeared in more than 1 list. A multicriteria decision-making (MCDM) Technique for Order of Preference by Similarity to Ideal Solution (TOPSIS) method^24^ was employed to determine relative opportunities for investment in biomedical product innovation by integrating measures of public health burden, cost, and current innovation activity for each pilot medical conditions. The TOPSIS method normalizes the measures and inputs each measure as a weighted variable to compute the relative goodness of a solution by estimating the distance between alternative solutions in a decision matrix. Domains were weighted equally with a value of 1. Quantitative scores for each of the 3 domains of public health burden, health care cost, and biomedical product innovation were calculated. Public health burden and health care cost scores were positive values, ie, higher scores contribute to a higher overall gap score, whereas biomedical product innovation was considered a negative value, with lower innovation scores contributing to a higher overall gap score. The ideal solution was defined as medical conditions with low public health burden, or low cost and high biomedical innovation. An overall gap score for each medical condition was calculated using TOPSIS and reflects the relative ranking compared with 1 extreme alternative (lowest possible score: a theoretical condition with the lowest public health burden, lowest cost, and highest innovation level) and the other extreme possible alternative (highest possible score: a theoretical condition with the highest public health burden, highest cost, and lowest innovation level). Low scores indicate an imbalance with relatively high investment in innovation relative to public health burden and cost, whereas high scores indicate an imbalance with relatively low investment in innovation relative to public health burden and cost (Figure 1). Medical conditions were rank ordered from highest to lowest overall gap score. The score interquartile range (IQR) was applied to classify the medical condition into high-, middle-, and low-score groups. Opportunities for action were identified by examining the relative contribution of scores from each domain to the overall gap score for each medical condition. Specifically, biomedical product innovation opportunities were defined as medical conditions in which the extent of biomedical product innovation activity lagged relative to the public health burden and health care cost. This study followed the Strengthening the Reporting of Observational Studies in Epidemiology (STROBE) reporting guidelines.^25^

## Results

**Figure 2.**
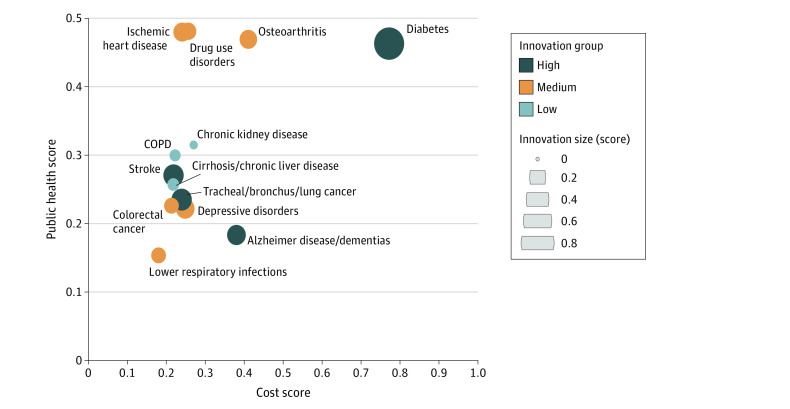
Multifactor Plot of Critical Biomedical Product Innovation Gaps The innovation score interquartile range was applied to classify the medical condition into high-, medium-, and low-innovation groups. Opportunities for action are identified by examining the relative contribution of scores from each domain to the overall gap score for each medical condition. Biomedical product innovation opportunities were defined as medical conditions in which the extent of biomedical product innovation activity lagged relative to the public health burden and health care cost. COPD indicates chronic obstructive pulmonary disease.

The 13 medical disorders selected for the pilot study and the associated *International Statistical Classification of Diseases and Related Health Problems, Tenth Revision (ICD-10)* codes are listed in eTable 1 in Supplement 1. The public health burden score, health care cost score, biomedical product innovation score, and overall gap score for each of the 13 identified medical conditions derived from the MCDM TOPSIS method are summarized in the Table and Figure 2.

**Table.** Composite Biomedical Product Innovation Gap, Public Health Burden, Health Care Cost, and Overall Gap Scores

Condition	Public health burden score^a^	Health care cost score^a^	Biomedical product innovation score^b^	Overall gap score^c^
Diabetes mellitus	0.46	0.77	0.77	0.61
Osteoarthritis	0.47	0.41	0.27	0.46
Drug use disorders	0.48	0.26	0.22	0.39
Ischemic heart disease	0.48	0.24	0.26	0.38
Alzheimer disease and other dementias	0.19	0.38	0.29	0.37
Chronic kidney disease	0.32	0.27	0.05	0.36
Chronic obstructive pulmonary disease	0.30	0.22	0.09	0.33
Cirrhosis and other liver disease	0.26	0.22	0.1	0.31
Colorectal cancer	0.23	0.22	0.14	0.30
Stroke	0.27	0.22	0.35	0.30
Depressive disorders	0.22	0.25	0.29	0.30
Tracheal, bronchus, and lung cancer	0.24	0.24	0.37	0.29
Lower respiratory infections	0.16	0.18	0.18	0.27

^a^ Positive score (higher score contributes to higher overall gap score).

^b^ Negative score (higher score contributes to lower overall gap score).

^c^ An overall gap for each medical condition score was calculated using the Technique for Order of Preference by Similarity to Ideal Solution method and reflects the relative ranking compared with 1 extreme alternative (lowest possible score: a theoretical condition with the lowest public health burden, lowest cost, and highest innovation level) and the other extreme possible alternative (highest possible score: a theoretical condition with the highest public health burden, highest cost, lowest innovation level). Low scores indicate an imbalance with relatively high investment in innovation relative to public health burden and cost, whereas high scores indicate an imbalance with relatively low investment in innovation relative to public health burden and cost.

### Public Health Burden Scores

The conditions with the greatest public health burden score in 2019 were ischemic heart disease (0.48), drug use disorders (0.48), and osteoarthritis (0.47). Although ischemic heart disease had the highest national mortality rate (170.03 per 100 000), drug use disorders had the highest YLD (911.11/100 000), and osteoarthritis had the highest prevalence (15 813.80/100 000). An analysis of longitudinal data between 2016 and 2019 confirmed that ischemic heart disease had the highest increase in mortality, whereas drug use disorders had the greatest increase in YLD, and osteoarthritis had the greatest increase in prevalence. The greatest disparities in mortality rates were observed in drug use disorders, with the non-Hispanic American Indian/Alaska Native population having the highest mortality rate (eTable 2 in Supplement 1).

### Health Care Cost Scores

Diabetes (0.77), osteoarthritis (0.41), and Alzheimer disease (0.38) had the highest health care cost scores in 2015. Patients with Alzheimer disease and other dementias had the highest OOP spending ($59.97 billion). Diabetes had the highest total public spending ($152.10 billion) and total spending ($171.51 billion). The greatest decrease in OOP costs between 2012 and 2015 was observed for ischemic heart disease, whereas the total public spending and total spending increased for osteoarthritis and diabetes, respectively (eTable 3 in Supplement 1).

### Biomedical Product Innovation Scores

The least amount of innovation was observed in chronic kidney disease (0.95), chronic obstructive pulmonary disease (COPD) (0.91), and cirrhosis and other liver diseases (0.90). eTable 4 in Supplement 1 details the specific innovation measure average values across the innovation pipeline for each condition from basic research to approval and market availability for the period from 2015 to 2019.

### Overall Gap Scores

Diabetes, osteoarthritis, and drug use disorders were the top 3 medical conditions with the highest overall gap score. The specific reason for the overall gap score was unique to each condition and reflects opportunities for action. For example, the high health care costs contributed to the overall gap score in diabetes; whereas the high public health burden score, due to the high prevalence, combined with high health care cost contributed to the overall gap score observed in osteoarthritis. The elevated overall gap score in drug use disorders was due to the high rates of disability and disparities, which increased the public health burden score.

## Discussion

We designed and pilot-tested a data-driven, quantitative model to identify, rank order, and prioritize opportunities for action by evaluating relative alignment between biomedical product innovation scores and measures of public health burden and health care cost for 13 medical conditions. This proof-of-concept study leverages a replicable framework to identify high-level gaps and opportunities that may be prioritized for investment by public health and industry leaders and stakeholders who fund research. Together with a granular analysis of potential reasons for the high-level gaps identified in specific medical disorders,^13^ our framework may inform resource allocation decisions to address these gaps.

Our model addressed the limitations of prior studies^6,7^ by analyzing measures both of public health burden and of health care costs. This pilot study used a quantitative process, leveraged input from a broad set of stakeholders, and elicited input from a variety of sources including an advisory board of experts. This proof-of-concept study demonstrates the feasibility of a data-driven model to identify relative gaps in innovation. Follow-up work may include creating a public process that emphasizes the involvement of a broad set of well-represented stakeholders while simultaneously continuing to ensure that the process of identifying gaps remains transparent. Further iterations of the model should consider using a Delphi process and incorporating weights into the TOPSIS model.

Although the overall gap score provided a high-level indicator, an in-depth analysis of the relative contribution of domain scores is a crucial step to guide targeted actions to address areas of highest need. For example, when potential reasons for why diabetes emerged as having the highest overall gap score were examined, health care costs, specifically public spending, was a primary driver. Because diabetes had the highest biomedical product innovation scores, allocating additional resources to this domain exclusively may not be effective in addressing the individual and health system burdens of this disorder. Although the current study did not examine the root causes of the high costs associated with diabetes, it is possible that an increase in the number of prescriptions of newer and innovative antidiabetic medications contributed to the increasing costs related to diabetes.^26,27,28,29,30,31^ This condition illustrates a paradox where high health care cost is a marker of public health need and burden, but increasing costs from innovation can create the appearance of an even higher need for innovation. Additional research must extend the gap scores generated by our model to target investments. Innovations in other aspects of health care, including optimizing health systems delivery, and implementation science may be required to improve outcomes in disorders with high public health burden and cost despite high levels of biomedical product innovation.

Our model may also identify focused opportunities to spur biomedical product innovation for specific medical conditions (Figure 2; eTable 2 in Supplement 1). A framework developed to identify potential reasons for gaps in the biomedical innovation ecosystem in the field of oncology can serve as a model for similar analyses for other medical disorders.^13,32^ Although colorectal cancer and lung cancer had very similar cost and public health burden scores, the NIH research funding allocation for colorectal cancer (approximately $346 million) was less than half the funding for lung cancer (approximately $850 million). This difference in NIH research funding may be related to the large differences seen in other innovation measures for these 2 cancers, including the number of patents (3200 vs 4513), late-stage venture capital funding ($33.9 million vs $179 million), and private deals ($551 million vs $4196 million). Chronic kidney disease, cirrhosis and other liver disease, and COPD had lower innovation investment relative to other conditions with similar or lower public health burden and health care cost and represent a significant opportunity. Although the federal government recently incentivized innovation in chronic kidney disease treatment,^33^ there is a need for improvements in the innovation pipeline for COPD as well as cirrhosis and other chronic liver diseases. The number of patents granted, early venture capital funding, NIH funding, and number of expedited approvals were significantly lower for COPD. Similarly, there were significant gaps in the number of early and late venture capital investments, private deals clinical trials, and number of devices and expedited approvals for cirrhosis.

Quantifying innovation and relative gaps in the biomedical ecosystem is a complex problem given the numerous interactions, functional areas, and long cycles in biomedical discovery and health care. The need to identify innovation gaps and opportunities concerning public health burden and health care cost is especially important given the long lead times typically required to bring discovery to market. The development of a new therapy, from target identification through marketing approval, on average takes over 12 years and frequently longer.^34^ Several regulatory options may help spur innovation in underinvested areas.^35,36^ By identifying gaps in specific phases of the biomedical innovation cycle, our findings may inform decisions to target resources, to include policies, to address the specific gap. Federal regulatory agencies can also enhance intellectual property (IP) protections (eg, longer-duration patent protection) for low-innovation, high-cost, high–public health burden medical disorders identified using our framework. Private industry can promote open innovation initiatives, where input for research and development is gathered from the public or through collaboration with other industries, in areas with the highest relative gap scores. Academic organizations could use the gap scores to inform faculty recruiting priorities and technology development priorities.

### Limitations

Although our framework considered public health burden, cost, and biomedical innovation, it did not include measures of barriers to the implementation of effective treatments such as workforce issues, health care system fragmentation that impedes access and continuity of care, and the extent of adoption and acceptance of evidence-based practices in health care. This proof-of-concept study was limited to a small subset of medical conditions. Had we included a larger set of conditions we may have identified other conditions with higher (or lower) gap scores. Data were obtained from multiple sources because all the data required for the study were not available in a single data repository. The IHME and NCHS differed in the methodology and timelines for data collection to include grouping of medical disorders (ie, Clinical Classification Software [CCS] codes, *ICD-10* codes). Notably, the IHME mortality data did not record deaths related to osteoarthritis and depression because they were considered risk factors, but not direct causes of death (for example, direct causes of deaths that may be related to these conditions include deaths from falls and suicides). Future work may incorporate downstream effects of risk factors on mortality, although reliable data across conditions may not exist. Incomplete data among some conditions within racial and ethnic distributions prevented a comprehensive assessment of health disparities. Although the data reflect the different stages of the biomedical product innovation pipeline, they do not provide detailed understanding of why specific phases are lagging. Furthermore, the narrow definition of biomedical product innovation used in the study did not include investments in innovations in health care delivery. Because replication of this model in other contexts requires access to robust health systems and data collection infrastructure, gaps identified in this study may mainly reflect priorities in the US and other high-income countries with requisite capabilities. Finally, it is important to note that IHME captured costs exclude spending on major investments such as hospital construction, health worker education and training, as well as research and development.

## Conclusions

In this cross-sectional pilot study, we developed and implemented a data-driven, proof-of-concept model that can help identify, quantify, and prioritize opportunities for biomedical product innovation. By simultaneously analyzing complementary measures across the health ecosystem, we identified medical conditions with the largest biomedical product innovation opportunities relative to public health burden and health care costs. With appropriate infrastructure, recent data, and automated data analytic techniques, this methodology can be refined and scaled to generate timely information to inform investments to improve public health across the nation.

## Notes

Supplement 1. eTable 1. List of Medical Disorders with associated International Classification of Diseases (ICD) codes37 Selected for the Pilot eTable 2. Final List of Conditions and Public Health Impact Metrics included in the TOPSIS Method eTable 3. Final List of Conditions and Healthcare Cost Metrics included in the TOPSIS Method eTable 4. Biomedical Product Innovation Pipeline Measures and Results (2015-2019)

Supplement 2. Data Sharing Statement
