# Supplementary material for: A Quantitative Framework to Identify and Prioritize Opportunities in Biomedical Product Innovation: A Proof-of-Concept Study
Source: JAMA Health Forum. 2023 May 5;4(5):e230894. doi: 10.1001/jamahealthforum.2023.0894 (PMC10163391; doi:10.1001/jamahealthforum.2023.0894)
Supplement: Supplement 1. — eTable 1. List of Medical Disorders with associated International Classification of Diseases (ICD) codes37 Selected for the Pilot eTable 2. Final List of Conditions and Public Health Impact Metrics included in the TOPSIS Method eTable 3. Final List of Conditions and Healthcare Cost Metrics included in the TOPSIS Method eTable 4. Biomedical Product Innovation Pipeline Measures and Results (2015-2019) [file jamahealthforum-e230894-s001.pdf]

Supplemental Online Content

Gressler LE, Crowley K, Berliner E, et al. A quantitative framework to identify and prioritize opportunities in biomedical product innovation: a proof-of-concept study. *JAMA Health Forum*. 2023;4(5):e230894. doi:10.1001/jamahealthforum.2023.0894

eTable 1. List of Medical Disorders with associated International Classification of Diseases (ICD) codes37 Selected for the Pilot
eTable 2. Final List of Conditions and Public Health Impact Metrics Included in the TOPSIS Method
eTable 3. Final List of Conditions and Healthcare Cost Metrics included in the TOPSIS Method
eTable 4. Biomedical Product Innovation Pipeline Measures and Results (2015-2019)

This supplemental material has been provided by the authors to give readers additional information about their work.

© 2023 Gressler LE, et al. *JAMA Health Forum.*

eTable 1. List of Medical Disorders with associated International Classification of Diseases (ICD) codes[37] Selected for the Pilot Study.

**Medical Disorders**	**ICD10**
Alzheimer's disease and other dementias	F00-F03.9, G30-G31.1, G31.8-G31.9
Chronic Kidney Disease	D63.1, E10.2, E11.2, I12-I13.9, N02-N08.8, N15.0, N18-N18.9, Q61-Q62.8
Chronic Obstructive Pulmonary Disease (COPD)	J40-J46.9
Cirrhosis and other chronic liver disease	B18-B18.9, I85-I85.9, I98.2, K70-K70.3, K71.7, K74-K74.9, K75.2, K75.4-K76.2, K76.4-K76.9, K77.8
Colorectal Cancer	C18-C21.9, D01.0-D01.3, D12-D12.9, D37.3-D37.5
Depressive disorders	F32-F33, F34.1
Diabetes Mellitus	E10-E10.1, E10.3-E11.1, E11.3-E11.9, P70.2, E12-E14
Drug Use Disorders	F11-F16.9, F18-F19.9, P04.4, P96.1, R78.1-R78.5, X40–X44, X60–X64, X85, Y10–Y14
Ischemic Heart Disease	I20-I25.9
Lower Respiratory Infections	A48.1, A70, B97.4-B97.6, J09-J15.8, J16-J16.9, J20-J21.9, P23.0-P23.4, U04-U04.9
Osteoarthritis	M15-M19
Stroke	G45-G46.8, I60-I63.9, I65-I66.9, I67.0-I67.3, I67.5-I67.6, I68.1-I68.2, I69.0-I69.3
Tracheal, Bronchus, and Lung Cancer	C33-C34.9, D02.1-D02.3, D14.2-D14.3, D38.1

© 2023 Gressler LE, et al. *JAMA Health Forum.*

eTable 2. Final List of Conditions and Public Health Impact Metrics included in the TOPSIS Method

	**PUBLIC HEALTH IMPACT METRICS**						
**Condition**	**Deaths (rate per 100K) (2019)**	**Slope Deaths (2015-2019)**	**YLD (rate per 100,000) (2019)**	**Slope YLD (2015-2019)**	**Prevalence (rate per 100,000) (2019)**	**Slope Prevalence (2015-2019)**	**Disparity (2019)**
Alzheimer's disease and other dementias	43.88	0.50	209.71	3.13	1494.82	25.46	2.40
Chronic Kidney Disease	32.61	0.31	177.99	0.03	12269.58	251.42	1.00
Chronic Obstructive Pulmonary Disease (COPD)	59.71	0.92	585.74	3.18	6143.06	66.79	1.00
Cirrhosis and other chronic liver disease	20.52	0.05	4.22	0.00	14355.81	106.72	1.00
Colorectal Cancer	25.62	0.68	36.59	7.95	419.78	1.14	3.79
Depressive disorders	0.00	0.00	808.75	-1.47	4662.03	-1.26	1.00
Diabetes / Diabetes Mellitus	23.70	0.56	896.94	19.68	11847.85	259.09	1.14
Drug Use Disorders	20.04	1.14	911.11	22.02	3262.36	48.68	7.03
Ischemic Heart Disease	170.03	2.87	90.40	1.02	2703.60	27.39	1.95
Lower Respiratory Infections	24.98	0.42	5.50	0.03	91.34	0.58	3.44
Osteoarthritis	0.00	0.00	605.63	18.52	15813.80	438.25	1.00
Stroke	57.76	0.83	331.43	7.46	2162.13	49.58	2.25
Tracheal, Bronchus, and Lung Cancer	62.87	1.21	18.86	0.32	135.40	2.37	2.09

© 2023 Gressler LE, et al. *JAMA Health Forum.*

eTable 3. Final List of Conditions and Healthcare Cost Metrics included in the TOPSIS Method

	**HEALTHCARE COST METRICS (7)**					
**Condition**	Mean Cost per Capita OOP in Billions (2016)	Slope Mean Cost per Capita OOP (2012-2016)	Mean Total Public Spending in Billions (2016)	Slope Mean Total Public Spending (2012-2016)	Mean Total Spending in Billions (2016)	Slope Mean Total Spending (2012-2016)
Alzheimer's disease and other dementias	59.97	-0.67	47.70	1.78	137.60	-0.57
Chronic Kidney Disease	1.86	0.00	22.98	1.23	36.07	2.46
Chronic Obstructive Pulmonary Disease (COPD)	6.40	-0.25	25.75	-0.20	74.13	1.94
Cirrhosis and other chronic liver disease	3.88	-0.17	56.73	-0.97	39.95	-0.12
Colorectal Cancer	1.24	-0.08	17.71	-0.40	13.56	-0.59
Depressive disorders	18.64	-0.67	78.81	-0.70	111.64	3.18
Diabetes / Diabetes Mellitus	20.79	0.74	152.10	16.85	171.51	15.95
Drug Use Disorders	3.34	0.04	7.82	0.18	27.63	1.17
Ischemic Heart Disease	9.80	-0.89	117.41	-2.60	149.30	0.18
Lower Respiratory Infections	6.36	-0.47	37.65	-0.49	55.63	2.57
Osteoarthritis	12.51	-0.07	122.95	3.72	112.40	5.51
Stroke	13.90	-0.46	42.89	1.55	73.08	0.80
Tracheal, Bronchus, and Lung Cancer	0.85	0.01	8.04	0.07	13.80	0.03

© 2023 Gressler LE, et al. *JAMA Health Forum.*

eTable 4. Biomedical Product Innovation Pipeline Measures and Results (2015-2019)

Early research — Clinical research — Product on market

	Innovation Measures (2015-2019)															
	Patents		Early-stage VC		Late-stage VC		NIH		Private Deals		Clinical Trials			# Approvals		
**Condition**	**App**	**Granted**	**#**	**$**	**#**	**$**	**#**	**$**	**#**	**$**	**Started**	**Ended**	**Results**	**Drug**	**Device**	**Expedited**
Alzheimer's disease and other dementias	3,435.4	3,080.0	20.4	240.6	18.4	195.6	3,930.6	2,106.7	164.4	3,522.8	145.8	106.8	34.6	0.0	0.0	0.0
Chronic Kidney Disease	731.6	605.0	3.4	35.7	2.4	73.0	719.4	286.2	14.8	410.7	112.0	102.4	37.8	1.0	1.0	0.0
Chronic Obstructive Pulmonary Disease (COPD)	1,394.8	1,220.0	7.4	33.3	5.4	74.4	547.4	254.8	43.8	576.0	187.8	185.6	61.6	1.0	3.0	0.0
Cirrhosis and other chronic liver disease	1,406.0	1,949.0	3.4	64.2	1.3	50.2	946.6	361.1	10.6	139.8	93.0	89.6	23.4	4.0	0.0	0.0
Colorectal Cancer	4,138.6	3,200.0	4.0	32.2	3.8	33.9	849.6	346.3	17.8	551.5	317.4	215.4	59.0	2.0	3.0	0.2
Depressive disorders	2,551.6	2,168.0	23.6	138.9	11.2	159.9	4,659.4	1,968.2	176.2	3,178.9	203.2	152.0	23.8	3.0	0.0	0.0
Diabetes / Diabetes Mellitus	10,427.0	7,703.0	58.6	571.3	51.0	1,029.2	5,417.2	2,570.6	421.8	12,659.9	856.6	805.2	120.8	4.0	12.0	1.2
Drug Use Disorders	606.6	1,919.0	3.8	38.8	2.4	37.3	4,149.4	2,004.6	58.4	1,816.0	437.8	428.4	143.0	1.0	0.0	1.2
Ischemic Heart Disease	4,705.8	4,513.0	2.6	25.6	2.7	52.1	2,185.4	987.4	14.6	227.1	238.0	215.8	82.0	3.0	37.0	0.0
Lower Respiratory Infections	606.6	707.0	13.6	53.1	8.0	95.0	2,355.0	1,685.6	30.8	299.9	291.6	171.6	33.8	4.0	3.0	1.2
Osteoarthritis	3,389.6	5,064.0	12.0	44.7	9.4	163.2	1,371.6	603.2	99.2	3,218.4	485.2	434.8	124.2	3.0	6.0	0.0
Stroke	5,000.0	6,979.0	24.0	111.7	19.0	182.1	3,712.0	1,863.7	160.8	1,439.2	591.0	494.0	81.2	0.0	0.0	0.0
Tracheal, Bronchus, and Lung Cancer	6,190.6	4,513.0	10.8	119.0	11.6	179.0	1,921.6	850.1	63.6	4,196.2	334.0	317.4	96.4	8.0	6.0	6.0

© 2023 Gressler LE, et al. *JAMA Health Forum.*
