# Supplementary material for: A Quantitative Framework to Identify and Prioritize Opportunities in Biomedical Product Innovation: A Proof-of-Concept Study
Source: JAMA Health Forum. 2023 May 5;4(5):e230894. doi: 10.1001/jamahealthforum.2023.0894 (PMC10163391; doi:10.1001/jamahealthforum.2023.0894)
Supplement: Supplement 2. — Data Sharing Statement [file jamahealthforum-e230894-s002.pdf]

Data Sharing Statement

Gressler. A Quantitative Framework to Identify and Prioritize Opportunities in Biomedical Product Innovation. *JAMA Health Forum*. Published May 05, 2023. doi:10.1001/jamahealthforum.2023.0894

Data
Data available: No
